# Similar Health Benefits of Endurance and High-Intensity Interval Training in Obese Children

**DOI:** 10.1371/journal.pone.0042747

**Published:** 2012-08-06

**Authors:** Ana Carolina Corte de Araujo, Hamilton Roschel, Andreia Rossi Picanço, Danilo Marcelo Leite do Prado, Sandra Mara Ferreira Villares, Ana Lúcia de Sá Pinto, Bruno Gualano

**Affiliations:** 1 University of Sao Paulo, School of Medicine – Division of Rheumatology, Sao Paulo, Sao Paulo, Brazil; 2 University of Sao Paulo, School of Physical Education and Sport – Laboratory of Nutrition and Metabolism Applied to Exercise, Sao Paulo, Sao Paulo, Brazil; 3 University of Sao Paulo, School of Medicine – Division of Endocrinology, Sao Paulo, Sao Paulo, Brazil; 4 University of Sao Paulo, School of Physical Education and Sport – Laboratory of Neuromuscular Adaptations to Strength Training, Sao Paulo, Sao Paulo, Brazil; University of Sao Paulo, Brazil

## Abstract

**Purpose:**

To compare two modalities of exercise training (i.e., Endurance Training [ET] and High-Intensity Interval Training [HIT]) on health-related parameters in obese children aged between 8 and 12 years.

**Methods:**

Thirty obese children were randomly allocated into either the ET or HIT group. The ET group performed a 30 to 60-minute continuous exercise at 80% of the peak heart rate (HR). The HIT group training performed 3 to 6 sets of 60-s sprint at 100% of the peak velocity interspersed by a 3-min active recovery period at 50% of the exercise velocity. HIT sessions last ∼70% less than ET sessions. At baseline and after 12 weeks of intervention, aerobic fitness, body composition and metabolic parameters were assessed.

**Results:**

Both the absolute (ET: 26.0%; HIT: 19.0%) and the relative VO_2_ peak (ET: 13.1%; HIT: 14.6%) were significantly increased in both groups after the intervention. Additionally, the total time of exercise (ET: 19.5%; HIT: 16.4%) and the peak velocity during the maximal graded cardiorespiratory test (ET: 16.9%; HIT: 13.4%) were significantly improved across interventions. Insulinemia (ET: 29.4%; HIT: 30.5%) and HOMA-index (ET: 42.8%; HIT: 37.0%) were significantly lower for both groups at POST when compared to PRE. Body mass was significantly reduced in the HIT (2.6%), but not in the ET group (1.2%). A significant reduction in BMI was observed for both groups after the intervention (ET: 3.0%; HIT: 5.0%). The responsiveness analysis revealed a very similar pattern of the most responsive variables among groups.

**Conclusion:**

HIT and ET were equally effective in improving important health related parameters in obese youth.

## Introduction

The incidence of juvenile obesity has dramatically increased worldwide in the last fifty years, mainly as a result of a physically inactive lifestyle and inappropriate diet habits [1], [2]. Not surprisingly, the most successful interventions aimed at preventing or treating obese children have primarily focused on physical fitness promotion, along with behavioral and nutritional counseling [1], [3], [4].

In spite of that, it is still unclear which type of exercise is capable of eliciting the greatest health benefits to obese children. Traditionally, the low-to-moderate-intensity endurance training (ET) has been the most common type of exercise recommended to improve body composition, physical capacity and overall health-related parameters (e.g., blood pressure, insulin resistance, lipid profile) in healthy and obese people [3], [4]. However, recently, a growing body of literature has also supported the efficacy of high-intensity interval training (HIT) in promoting health-related effects in healthy children [5], [6] and adults [7], [8], and individuals with metabolic syndrome [9] and congestive heart failure [10].

The HIT consists of high-intensity exercise bouts interspersed by an interval period between the sets. The claimed advantage of HIT relies in the fact that this type of training is less-time consuming than ET, while producing comparable beneficial adaptations. For instance, Tjønna et al. [9] demonstrated that a 16-week, three times a week HIT (i.e., four 4-minute bouts at 90% of maximal heart rate with a 3-minute active recovery) and ET (i.e., 47 minutes at 70% of maximal heart rate) programs were equally effective in lowering mean arterial blood pressure and reducing body mass and fat in metabolic syndrome patients. Nonetheless, HIT was superior to ET in enhancing endothelial function, skeletal muscle biogenesis, and excitation-contraction coupling and in reducing blood glucose and lipogenesis in adipose tissue. Using a similar approach, Wisløff et al. [10] found similar results in heart failure patients. Peak oxygen consumption (VO_2peak_) and endothelial function improved more with HIT than moderate ET and was associated with reverse left ventricular remodeling. Moreover, quality of life was equally improved in both groups. In healthy young subjects, serial studies by Gibala's laboratory have showed that HIT (i.e., six repeats of a 30-s all-out Wingate Test with 4.5 min recovery between repeats) and ET (i.e., 40–60 min of continuous cycling at a workload that elicited ∼65% of VO_2peak_) induce similar metabolic, cardiovascular and skeletal muscle molecular adaptations in healthy humans [7], [11]. In a recent review, Gibala and Mcgee [8] stated that in young healthy persons of average fitness, HIT is a time-efficient strategy to stimulate a number of skeletal muscle adaptations that are comparable to traditional ET. The authors, however, stressed the fact that “fundamental questions remain regarding the minimum volume of exercise necessary to improve physiological well-being in various populations and the effectiveness of alternative (less extreme) interval-training strategies”.

Prolonged physical activities (i.e., >30 min) are contrary to a child's pattern of spontaneous exercise, which mainly comprises short-term intermittent efforts [12], [13]. It is well-known that children usually present higher perceived exertion in response to prolonged exercise [14]. This, along with psychological and cultural factors (e.g., shorter attention span, the need for recreational simuli, or motivation), may explain a child's preference for activities of shorter duration. Therefore, HIT emerges as a promising time-efficient and more motivational strategy capable of promoting health adaptions in children. In fact, a few studies have corroborated the potential of HIT training in inducing cardiovascular adaptations in healthy pediatric populations [15], but none of them have compared the efficacy of HIT *versus* ET in physically-inactive obese youth.

Given that exercise has been recognized as a major cornerstone of juvenile obesity management, efforts focused on determining the ideal type of training for treating this condition are of great relevance. Thus, the aim of this study was to compare two modalities of exercise training (i.e., HIT and ET) on health-related parameters in obese children.

## Materials and Methods

### Study sample and experimental design

One hundred outpatients were recruited from the Obesity Clinics (Endocrinology Department, School of Medicine, University of Sao Paulo, Brazil). Sixty-one did not meet inclusion criteria. Thirty-nine consecutive outpatient children were selected. The inclusion criteria were as follows: (1) age between 8 and 12 years; (2) BMI = 95^th^ percentile, according to the First National Health and Nutrition Examination Survey; (3) no pharmacological treatment; (4) no evidence of metabolic, hormonal, orthopedic, and cardiovascular disease at the time of the study's commencement; and (5) no participation in any regular exercise training program (except physical education classes, two days a week) at least 6 months before the commencement of the study and throughout the protocol. Children were randomly allocated into 2 groups: endurance training (ET) and sprint interval training (HIT). Subjects' demographic characteristics are presented in Table 1.

**Table 1:** Main patients' characteristics.

	ET	HIT	P
**Gender, F∶M**	11∶4	10∶5	-
**Age, yrs**	10.4 (0.9)	10.7 (0.7)	0.92
**Pubertal stages I/II/III/IV**	5/5/0/5	6/4/1/4	-
**Weight, kg**	67.9 (16.5)	73.7 (10.8)	0.65
**Height, cm**	150.4 (10.5)	153.6 (7.5)	0.76
**Body mass index, kg/m^2^**	29.6 (4.0)	30.8 (3.7)	0.81

Data are expressed as mean (sd). Abbreviations: HIT = endurance training; ET = endurance training.

At baseline (PRE) and after 12 weeks (POST) of exercise training, aerobic fitness, body composition and metabolic parameters were assessed. Food intake was assessed at PRE and POST, but no dietetic intervention was implemented. Throughout the study, the children and their parents received generic counseling by a professional nutritionist regarding the benefits of adopting healthy eating patterns. Children were submitted to medical examination on a weekly basis and possible adverse events were recorded.

The protocol was approved by the local Ethics Committee (General Hospital, School of Medicine, University of Sao Paulo) and written consent was obtained from all patients' parents at the beginning of the study.

### Pubertal evaluation

Pubertal developmental stage was determined according to the methods described by Marshall and Tanner [16], [17].

### Anthropometric measurements

Body mass was measured by an electronic body mass scale with children dressed in a light T-shirt and shorts. Height was measured by a stadiometer. Waist circumference was measured at the level of the umbilicus, using a non-stretchable tape.

### Bioelectrical impedance

Body composition was analyzed by bioelectrical impedance method using a standardized body composition analyzer (BiaQuantum RJL Systems, Inc, MI, EUA). Percentage of body fat was estimated using a validated equation adjusted for gender, age, weight, and height, following previously described procedures [18]. Children were instructed to refrain from drinking and eating for 4 hours before the test and exercising for at least 12 hours before the test.

### Arterial Pressure

Sitting systolic and diastolic blood pressure (SBP and DBP, respectively) were recorded following standard procedures and using a mercury column sphygmomanometer, after a 5-min period of absolute rest and with the patient seated.

### Metabolic parameters

Blood samples were collected through a catheter inserted into the antecubital vein after a 12-hour fasting period. Serum glucose, insulin, glycated hemoglobin (Hb1aC), leptin, total cholesterol and sub-fractions (i.e., LDL-, HDL- and VLDL-cholesterol) and triglyceride (TG) were measured in the clinical laboratory of the General Hospital (School of Medicine, University of Sao Paulo, Brazil) using standardized methods. The Homeostasis Model Assessment for Insulin Resistance (HOMA-IR) was calculated using the following equation: (insulin resistance = insulin (µU/ml)×glucose (mmol/L)/22.5).

### Maximal graded cardiorespiratory test

A modified Balke treadmill (Centurion, model 200, Micromed, Brazil) test was performed. Oxygen consumption (VO_2_) and carbon dioxide output (CO_2_) were obtained through breath-by-breath sampling and expressed as a 30-second average using an indirect calorimetry system (Cortex - model Metalyzer III B, Leipzig, Germany). Heart rate (HR) was continuously recorded at rest, during exercise and at recovery, using a 12-lead electrocardiogram (Ergo PC Elite, InC. Micromed, Brazil). The recovery period was set at four minutes using the initial workload (1.9 mph). Peak oxygen consumption (VO_2peak_) and ventilatory threshold (VT) and respiratory compensation point (RCP) were determined according to previously described criteria [19]. Time-to-exhaustion was recorded for each test.

Additionally, HR recovery (ΔHRR) was defined as the difference between HR at peak exercise and at both first (ΔHRR1) and second (ΔHRR2) minutes after exercise.

### Food intake assessment

Food intake was assessed at PRE and POST by three 24-h dietary recalls undertaken on separate days (2 weekdays and 1 weekend day). To help subjects estimate portion sizes, a visual aid photo album of real foods (Portion Photos of Popular Foods, 1997 - The American Dietetic Association, Chicago, IL, USA) and real-size three-dimensional fake food samples (TBW, São Paulo, SP, Brazil) were used. The 24-h dietary recall consists of the listing of all foods and beverages consumed during the 24 h prior to the recall. Energy and macronutrient intake were analyzed by the software Dietpro 5.1 Professional.

### Training Protocols

The training protocols consisted of walking/running exercise on a treadmill (Centurion, model 200, Micromed, Brazil) performed twice a week on alternate days for 12 weeks.

The ET group performed a 30-minute continuous endurance exercise at 80% of the peak HR. Training progression in the ET group was applied by increasing the exercise duration by 10 minutes every three weeks, until a total of 60 minutes during the last three weeks of intervention (i.e., from weeks 10 to 12). HR was continuously monitored during exercise to ensure that the subjects trained at the target intensity. The ET energy cost estimated by indirect calorimetry ranged from 268.1±61.4 to 536.2±122.8 Kcal, considering 30 and 60 minutes of exercise, respectively.

The HIT protocol consisted of repeated 60-second efforts (covered distance per bout: 118±14.5 m) at 100% of the peak velocity (determined by the maximal graded cardiorespiratory test), interspersed by a 3-min active recovery period at 50% of the peak velocity. Training progression was applied by adding one bout of exercise every three weeks. The number of bouts ranged from 3 (within the first 3 weeks) to 6 (within weeks 10–12). The HIT energy cost (recovery periods included) ranged from 84.0±1 5.3 to 169.7±30.6 Kcal, considering 3 and 6 bouts of exercise, respectively.

After 6 weeks of training, a new maximal graded cardiorespiratory test was conducted for training intensity adjustments.

### Statistical Analysis

After the normality and homogeneity of the variance were confirmed, the dependent variables were compared using a mixed model analysis with repeated measures (SAS 8.2, SAS Institute Inc., Cary, NC, USA) assuming group and time as the fixed factors and subjects as the random factor. A post hoc test adjusted by Tukey was used for multicomparison purposes. Significance level was previously set at p<0.05. Data are presented as mean ± standard deviation. Additionally, we performed a responsiveness analysis. To that end, commonly used indices of responsiveness (standardized response mean (SRM), Cohen's effect size (ES), percent change from baseline, and p values from the mixed model analysis) were used. The SRM was calculated by dividing the mean change in scores by the standard deviation of the change whereas the ES was calculated by dividing the mean change by the standard deviation of the baseline value (PRE) for each parameter. Finally, we calculated an overall rank of responsiveness. The rank was computed based on the sum score for all 4 responsiveness statistics. Only the parameters that showed statistical significance were included in the rank analysis.

## Results

**Figure 1:**
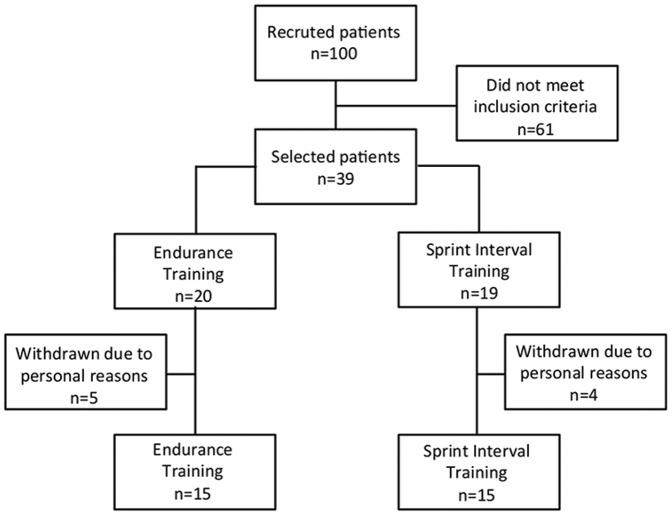
Fluxogram of patients.

The number of patients recruited to the study is shown in Figure 1. All of the 100 volunteers who responded to the initial request were screened and 39 met the inclusion criteria. These patients were randomly assigned to either the ET (n = 20) or HIT (n = 19) groups. Nine patients withdrew from the study for personal reasons (five from the ET group and four from the HIT group). Therefore, 30 patients were analyzed (ET = 15; HIT = 15). The adherence to the training program was similar between groups (85.5 and 86.9%, for the ET and HIT, respectively). Food intake remained unchanged after the intervention (Total energy intake – ET PRE: 1925 POST: 1893 kcal; HIT PRE: 2380 POST: 2365 kcal; Carbohydrate intake – ET PRE: 50.1 POST: 49.9%; HIT PRE: 48.3 POST: 43.0%; Protein intake – ET PRE: 19.0 POST: 19.6%; HIT PRE: 16.4 POST: 15.5%; Lipid intake – ET PRE: 30.7 POST: 30.0%; HIT PRE: 35.2 POST: 41.5%; p>0.05 for within- and between-group comparisons).

**Figure 2:**
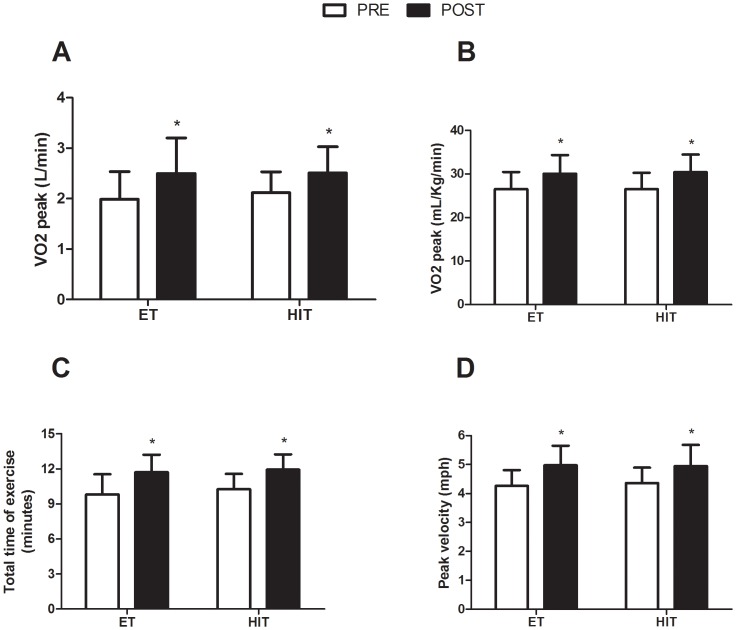
Effects of ET and HIT on cardiorespiratory and exercise parameters in response to a maximal graded exercise test. Panel A: VO_2_ peak (L/min); Panel B: VO_2_ (ml/kg/min); Panel C: Total time of exercise (min); Panel D: Peak velocity (mph); ET = endurance training group; HIT = high-intensity interval training; PRE = baseline; POST = after twelve weeks of training. * indicates p<0.05 (within-group comparison).

Both the absolute (ET: 26.0%; HIT: 19.0%) and the relative VO_2_ peak (ET: 13.1%; HIT: 14.6%) were significantly increased in both groups after the intervention. Additionally, the total time of exercise (ET: 19.5%; HIT: 16.4%) and the peak velocity during the maximal graded cardiorespiratory test (ET: 16.9%; HIT: 13.4%) were significantly improved across interventions (Figure 2).

**Figure 3:**
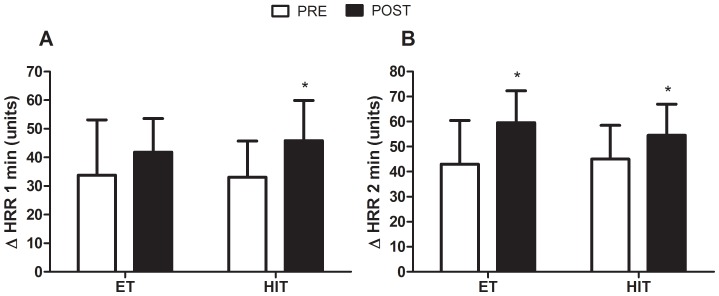
Effects of ET and HIT on absolute changes in heart-rate recovery. Panel A: absolute changes at the first (Δ HRR1 min) minute of recovery after a maximal graded exercise test at baseline (PRE) and after twelve weeks of training (POST). Panel B: absolute changes at the second (Δ HRR2 min) minute. ET = endurance training group; HIT = high-intensity interval training; * indicates p<0.05 (within-group comparison); # indicates main time effect (p<0.05).

ΔHRR1 and ΔHRR2, which are markers of aerobic fitness and autonomic function, were significantly increased in the HIT group (38.5 and 21%, respectively), whereas only deltaHRR2 increased in the ET group (38.8%) (Figure 3).

Insulinemia (ET: 29.4%; HIT: 30.5%) and HOMA-index (ET: 42.8%; HIT: 37.0%) were significantly lower for both groups at POST when compared to PRE. The other biochemical parameters remained unchanged in both groups (Table 2).

**Table 2:** Effects of ET and HIT on anthropometric measurements and arterial blood pressure in obese children.

*Variable*	PRE	POST	p (within-group comparison)	ES
Fat mass (%)				
*ET (n = 15)*	37 (4)	36 (4)	0.980	−0.150
*HIT (n = 15)*	38 (5)	37 (4)	0.680	−0.048
Fat mass (Kg)				
*ET (n = 15)*	25 (8)	25 (8)	0.990	0.031
*HIT (n = 15)*	28 (7)	27 (6)	0.540	−0.191
FFM (%)				
*ET (n = 15)*	63 (4)	64 (4)	0.990	0.157
*HIT (n = 15)*	63 (5)	62 (6)	0.820	−0.240
FFM (Kg)				
*ET (n = 15)*	43 (10)	43 (9)	1.000	−0.035
*HIT (n = 15)*	46 (6)	43 (4)	0.280	−0.473
Weight (Kg)				
*ET (n = 15)*	68 (16)	67 (16)	0.600	−0.050
*HIT (n = 15)*	74 (10)	72 (10)	0.030*	−0.182
Height (cm)				
*ET (n = 15)*	150 (0)	151 (0)	0.001*	0.128
*HIT (n = 15)*	152 (0)	154 (0)	0.008*	0.269
BMI (Kg/m^2^)				
*ET (n = 15)*	30 (4)	29 (4)	0.003*	−0.213
*HIT (n = 15)*	32 (3)	30 (3)	0.0001*	−0.454
Waist circumference (cm)				
*ET (n = 15)*	99 (10)	92 (9)	0.080	−0.610
*HIT (n = 15)*	99 (10)	96 (8)	0.290	−0.280
SBP (mmHg)				
*ET (n = 15)*	110 (9)	110 (13)	0.400	0.021
*HIT (n = 15)*	115 (10)	106 (10)	0.020*	−0.896
DBP (mmHg)				
*ET (n = 15)*	66 (10)	61 (6)	0.140	−0.532
*HIT (n = 15)*	66 (8)	62 (6)	0.330	−0.462

Data are expressed as mean (sd). Abbreviations: ET = endurance training; HIT = high-intensity interval training; FFM = free fat mass; BMI = body mass index; SBP = systolic blood pressure; DBP = diastolic blood pressure; ES = effect size.

Body composition parameters are shown in Table 3. Body mass was significantly reduced in the HIT (2.6%), but not in the ET group (1.2%). Despite of the slight reduction in body mass observed in the HIT group, no between-group differences were found at the POST test. A significant reduction in BMI was observed for both groups after the intervention (ET: 3.0%; HIT: 5.0%).

**Table 3:** Effects of ET and HIT on metabolic parameters in obese children.

*Variable*	PRE	POST	p (within-group comparison)	ES
Glycemia (mg/dL)				
*ET (n = 15)*	92 (12)	88 (7)	0.140	−0.360
*HIT (n = 15)*	92 (6)	89 (6)	0.550	−0.510
Insulinemia (µU/mL)				
*ET (n = 15)*	22(11)	16 (8)	0.008*	−0.600
*HIT (n = 15)*	21 (9)	15 (6)	0.010*	−0.700
Total cholesterol (mg/dL)				
*ET (n = 15)*	156 (26)	164 (30)	0.400	0.300
*HIT (n = 15)*	164 (28)	165 (34)	0.880	0.050
HDL-C (mg/dL)				
*ET (n = 15)*	43 (10)	46(11)	0.710	0.210
*HIT (n = 15)*	43 (6)	46 (7)	0.730	0.370
LDL-C (mg/dL)				
*ET (n = 15)*	94 (21)	100 (22)	0.560	0.270
*HIT (n = 15)*	102 (24)	104 (30)	0.850	0.080
VLDL-C (mg/dL)				
*ET (n = 15)*	18 (8)	19 (7)	0.990	0.008
*HIT (n = 15)*	19 (5)	17 (6)	0.800	−0.330
Triglycerides (mg/dL)				
*ET (n = 15)*	93 (39)	93 (37)	0.990	−0.001
*HIT (n = 15)*	93 (25)	84 (32)	0.750	−0.350
HOMA-index				
*ET (n = 15)*	5 (3)	3 (2)	0.006*	−0.730
*HIT (n = 15)*	5 (2)	3 (2)	0.002*	−0.700
Glycated hemoglobin				
*ET (n = 15)*	5 (0)	5 (0)	0.490	−0.510
*HIT (n = 15)*	5 (0)	5 (0)	0.990	0.020
Leptinemia (mg/dL)				
*ET (n = 15)*	43 (19)	36 (15)	0.210	−0.380
*HIT (n = 15)*	47 (14)	43 (16)	0.740	−0.270

Data are expressed as mean (sd). Abbreviations: ET = endurance training; HIT = high-intensity interval training; ES = effect size.

The responsiveness statistics indicate the cardiorespiratory variables as the most responsive parameters following the intervention. Amongst the five top-ranked variables, three were related to the aerobic fitness. The remaining variables were related to the glucose metabolism (i.e., HOMA-index and insulinemia). In general, both groups presented rather similar parameters in the total ranking (Table 4).

**Table 4:** Ranking of the studied parameters.

	P (rank)		ES (rank)		SRM (rank)		Delta (rank)		Total rank	
	*ET*	*HIT*	*ET*	*HIT*	*ET*	*HIT*	*ET*	*HIT*	*ET*	*HIT*
**Time-to-exhaustion**	0.0001 (1)	0.0002 (2)	1.1 (2)	1.3 (1)	1.5 (1)	1.1 (1)	18.5 (4)	19.2 (4)	1	1
**Absolute VO2 peak**	0.004 (5)	0.02 (7)	0.9 (3)	0.9 (4)	0.9 (4)	0.8 (4)	23.1 (3)	26.3 (3)	4	5
**Relative VO2 peak**	0.001 (2)	0.004 (4)	0.9 (3)	1.0 (3)	1.2 (3)	0.7 (5)	17.8 (5)	13.3 (7)	2	6
**Peak velocity**	0.002 (3)	0.001 (5)	1.3 (1)	1.1 (2)	0.9 (4)	1.0 (2)	15.0 (6)	16.2 (5)	3	3
**HOMA**	0.006 (6)	0.002 (3)	−0.7 (4)	−0.8 (5)	−0.9 (4)	−0.9 (3)	−28.4 (1)	−31.3 (1)	4	2
**Insulinemia**	0.009 (7)	0.01 (6)	−0.6 (5)	−0.7 (6)	−0.8 (5)	−1.0 (2)	−26.4 (2)	−28.3 (2)	5	4
**Glicemia**	0.140 (9)	0.55 (11)	−0.4 (7)	−0.5 (7)	−0.3 (8)	−0.5 (6)	−2.7 (10)	−5.4 (11)	10	11
**Leptinemia**	0.210 (11)	0.74 (13)	−0.4 (7)	−0.3 (8)	−0.2 (9)	−0.5 (6)	−7.4 (7)	−14.6 (6)	8	9
**BMI**	0.003 (4)	0.0001 (1)	−0.2 (8)	−0.5 (7)	−1.3 (2)	−0.8 (4)	−5.0 (8)	−2.9 (14)	6	7
**Waist circumference**	0.080 (8)	0.29 (9)	−0.6 (5)	−0.3 (8)	−0.4 (7)	−0.8 (4)	−3.6 (9)	−4.0 (13)	7	10
**Weight**	0.600 (13)	0.03 (8)	−0.1 (9)	−0.2 (9)	−0.7 (6)	−0.3 (8)	−2.5 (12)	1.2 (15)	11	12
**SBP**	0.400 (12)	0.02 (7)	0.0 (10)	−0.9 (4)	−0.8 (5)	0.0 (9)	−6.6 (8)	−5.8 (10)	10	8
**DBP**	0.140 (9)	0.33 (10)	−0.5 (6)	−0.5 (7)	−0.2 (9)	−0.3 (8)	−3.0 (10)	−6.6 (9)	8	10
**HR recovery**	0.150 (10)	0.73 (12)	−0.7 (4)	−0.3 (8)	−0.4 (7)	−0.4 (7)	−2.4 (13)	−4.9 (12)	8	12

Details on ranking analysis can be found in **Statistical Analysis.** Abbreviations: ET = endurance training; HIT = high-intensity interval training; SRM = standardized response mean; ES = effect size; VO2 = oxygen consumption; BMI = body mass index; SBP = systolic blood pressure; DBP = diastolic blood pressure; HR = heart rate.

Based upon the medical examination, no clinical evidence of excessive exhaustion, pain, osteoarticular injury, muscle soreness, or any other adverse event was noticed.

## Discussion

The main focus of this study was to compare the effects of ET *versus* HIT on health-related parameters in obese youth. We demonstrated for the first time that the both types of training were equally effective in improving metabolic parameters, BMI, and aerobic fitness in this sample.

Along with dietetic counseling, exercise has been considered the major cornerstone of juvenile obesity management [1], [3], [4]. However, the optimal type of training capable of eliciting the most important health benefits to obese children remains debatable. In this context, HIT has been recently suggested as an alternative method to ET for metabolic and cardiovascular status improvement for broad populations, from young health adults [11] to old heart failure patients[10]. In health pediatric populations, HIT has been shown to improve VO_2peak_ [5], [15], [20], [21], maximal velocity in the incremental test [5], high-intensity intermittent performance[15], peak and submaximal oxygen pulse [20], and resting pulmonary function and ventilatory response to exercise [22]. In light of these previous findings, the novelty of the current study was two-fold: 1) the investigation of physically-inactive obese children; and 2) the evaluation of other health-related parameters (e.g., insulin resistance and body composition measures) in addition to performance-related variables in a pediatric sample.

The present results confirm the efficacy of HIT (to the same extent as ET) in improving aerobic fitness (e.g., VO_2peak_, time-to-exhaustion) in pediatric populations, extending this notion to obese youth. Interestingly, HR recovery, which is significantly delayed in juvenile obesity [23], was also improved following both HIT and ET, further supporting the therapeutic role of exercise in improving physical fitness and autonomic function, irrespective of the training modality.

Furthermore, both exercise modes were equally and substantially effective in improving insulinemia and HOMA-index, which are surrogate markers of insulin sensitivity. However, the other biochemical parameters remained stable. In this respect, it is worth noting that the individuals presented metabolic parameters within a desirable range, which might explain the lack of changes in potentially modifiable factors, such as lipid profile.

With exception of SBP and body mass which were attenuated solely in the HIT group (within-group comparisons), both HIT and ET were equally effective in promoting health-related effects. This was further confirmed by a comprehensive responsiveness analysis based on the rank tests, which revealed a very similar pattern among groups of the most responsive variables, with those related to aerobic fitness and insulin sensitivity being situated in the top-five rank. Collectively, the present data allow concluding that both HIT and ET may be used as an efficient and safe strategy to improve health in obese youth. However, one must be aware that children may be naturally prone to short bouts of intensive exercise rather than prolonged continuous exercise [12]. Moreover, it is important to emphasize that HIT sessions was substantially less time-consuming (∼70%) than ET sessions. Thus, the fact that HIT is a time-efficient strategy that meets the child's preference of physical fitness may be considered an advantage of this exercise type over ET. Long-term studies should verify whether HIT training does confer more beneficial results in terms of adherence and consequently health outcomes in comparison with ET.

This study presents some limitations. First, a control group was not included. Children were recruited from a medical hospital where they receive multidisciplinary treatment for obesity. Given that exercise is the first line treatment for juvenile obesity, it would be ethically unacceptable to have our outpatients refrain from exercise. Notwithstanding this recognized limitation, it is important to stress the short-term characteristic of this study, mitigating the impact of the maturation on the study's outcomes and, hence, the lack of the non-trained group. In support of this, none of the individuals had the sexual maturation status changed after the intervention. Second, the short-term follow-up itself is another limitation, precluding definitive conclusions regarding the safety and efficacy of the interventions. Finally, to allow clearly distinguishing the effect of the training, exercise training was not accompanied by any dietetic prescription or psychological therapy. Further studies should investigate the possible synergistic effect of these types of training in addition to non-pharmacological interventions.

Exercise training is one of the most efficient strategies in the treatment of juvenile obesity. However, little is known on the differential effects yielded by alternative training protocols. In this respect, the current study provided evidence that HIT may be as effective as traditional ET in improving general health parameters in obese children. From a practical standpoint, HIT may be incorporated into therapeutic programs aimed to treat juvenile obesity, since this mode of exercise is less-time consuming and probably more pleasant to children population. From a scientific perspective, however, one should be aware that there are questions remaining on this topic still to be elucidated, such as: *“Does the efficacy of HIT hold true on a long-term basis?”*, *“Is HIT as safe as ET in obese children?”*, *“Do obese children really prefer HIT over ET?”*, “*Do energy expenditure-matched HIT and ET programs produce comparable health benefits?”.* Further studies with large and diversified cohort of obese children will be necessary to address these questions and advance our knowledge on this emerging type of training.

In conclusion, both HIT and ET were equally effective in improving important health parameters (e.g., aerobic fitness, insulin sensitivity, BMI) in obese children. In light of the equivalence of HIT and ET, the former emerges as a novel time-efficient and potentially motivational strategy capable of promoting health adaptations in juvenile obesity.
